# The impact of perioperative enhanced recovery nursing model on postoperative delirium and rehabilitation quality in elderly patients with femoral neck fractures

**DOI:** 10.1186/s12891-023-07068-4

**Published:** 2023-12-06

**Authors:** Cheng Wang, Bingyin Tan, Qing Qian

**Affiliations:** 1 Department of Orthopedics, Wuhan Fourth Hospital, No. 473, Hanzheng Street, Qiaokou District, Wuhan, 430033 China; 2 Department of Pharmacy, Wuhan Fourth Hospital, No. 473, Hanzheng Street, Qiaokou District, Wuhan, 430033 China

**Keywords:** Enhanced recovery after surgery, Nursing model, Delirium, Sleep quality, Femoral neck fractures

## Abstract

**Background:**

The aim of this study was to investigate the effects of introducing the Enhanced Recovery After Surgery (ERAS) nursing model on postoperative delirium occurrence and rehabilitation quality in elderly patients with femoral neck fractures.

**Methods:**

A total of 160 elderly patients with femoral neck fractures, who met the inclusion criteria and were admitted between March 2021 to March 2023, were divided into two groups: the traditional care group and the ERAS nursing model group. In addition to traditional care measures, the ERAS nursing model group received interventions based on the principles of the ERAS nursing model. The occurrence of delirium and sleep quality were observed at 24, 48, and 72 h postoperatively, as well as during the overall hospital stay. The duration of hospitalization, time to first mobilization, and post-discharge follow-up on quality of life were compared between the two groups.

**Results:**

The ERAS nursing model group exhibited a significant difference in the occurrence of delirium at 48 and 72 h postoperatively, as well as during the overall hospital stay (*P* < 0.05). However, there was no significant difference in the occurrence of delirium at 24 h postoperatively (*P* > 0.05). The sleep quality of the two groups showed a statistically significant difference (*P* < 0.05). The ERAS nursing model group had shorter time to first mobilization, reduced hospitalization duration, and higher Harris and SF-36 scores during post-discharge follow-up, compared to the traditional care group (*P* < 0.05).

**Conclusions:**

The implementation of the ERAS nursing model in elderly patients with femoral neck fractures improved postoperative sleep quality, reduced delirium occurrence, shortened average hospitalization duration, and enhanced patients’ quality of life.

## Introduction

Femoral neck fractures (FNF) remain a considerable public health issue, both from a clinical and a socio-economic standpoint. This type of fracture is a paradigmatic subtype of hip fractures and frequently results from altered biomechanical constructs under the influence of various factors. FNF accounts for approximately 3.59% of all fractures globally, making it a pressing concern in the field of orthopedic medicine [1, 2]. Total hip arthroplasty (THA) is a prevalent surgical intervention for FNF and is usually the treatment of choice for most clinicians. However, patients undergoing THA are at risk for postoperative complications, one of the most consequential being delirium. Delirium, an acute onset of consciousness disorder mainly characterized by attention deficits and perceptual abnormalities, is a considerable challenge in the postoperative management of FNF patients [3, 4].

Delirium’s exact etiology remains elusive. Despite its reversibility, the long-term cognitive impairments linked to prolonged delirium episodes can persist for months, morphing into a chronic condition. This is particularly problematic in the elderly population, where the likelihood of postoperative delirium is markedly high [4]. Studies indicate a postoperative delirium incidence of up to 44% among elderly FNF patients. Alarmingly, the mortality rate is about three times higher in those who experience delirium compared to those who do not [5]. The scholarly consensus highlights the importance of preventing delirium, which far outweighs the value of treating it once it has occurred. The prevention-first approach underscores the crucial role of comprehensive and diligent perioperative nursing care for patients undergoing surgery for FNF.

Enter the Enhanced Recovery After Surgery (ERAS) nursing model. This modern approach to perioperative care integrates a suite of evidence-based interventions aimed at expediting patient recovery [5]. ERAS has seen increasing adoption across various clinical disciplines, with impressive results attesting to its effectiveness. Evidence suggests that employing the ERAS nursing model can considerably mitigate postoperative complications in hip fracture patients, reducing the duration of hospital stays [6]. Given these promising outcomes, our institution decided to implement the ERAS model for postoperative care in elderly FNF patients. We have observed satisfactory clinical results that align with the literature findings.

The application and success of the ERAS nursing model in various clinical settings have been well-documented, as evinced by recent studies such as those by Jiang et al. and Kim et al. [7, 8]. The aim of this study is to delve deeper into the impact of the ERAS nursing model on postoperative delirium and the quality of rehabilitation in elderly patients with FNF. By providing a robust investigation of this intervention, we hope to reinforce the importance of systematic, evidence-backed approaches in perioperative care. We believe that the successful implementation of the ERAS model will result in improved patient outcomes, a more swift and effective return to normalcy for patients, and an overall decrease in healthcare burdens. Our ultimate goal is to advocate for the wider adoption of this model in managing FNF patients, thereby enhancing their postoperative quality of life and overall survival outcomes.

## Methods

### Study design

A total of 160 elderly patients who underwent total hip arthroplasty (THA) for femoral neck fractures at our orthopedic department from March 2021 to March 2023 were selected as the research subjects. Inclusion criteria were as follows: (1) Age ≥ 65 years; (2) Clear diagnosis of unilateral femoral neck fracture; (3) Fracture classified as Garden type III or IV [9]; (4) Total hip arthroplasty was the chosen surgical method; (5) Absence of significant mental illness; (6) Willingness to participate in this study. Exclusion criteria included: (1) Cognitive impairment; (2) History of cerebral infarction, cerebral hemorrhage, traumatic brain injury, and psychiatric disorders; (3) Abnormalities in cardiac and renal function; (4) Abnormal blood glucose control; (5) Inability to cooperate with follow-up or incomplete follow-up data. The study was approved by the hospital’s Ethics Committee. The 162 patients who met the criteria were selected chronologically by admission date. From March 2021 to March 2023, 75 patients were placed in the traditional care group, and from March 2021 to March 2023, 87 patients were placed in the ERAS nursing model group. Two patients from the traditional care group, who developed suspected venous thrombosis 4 days post-surgery, was referred to an external hospital and dropped out of the study. The final study population consisted of 73 patients in the traditional care group and 87 patients in the ERAS nursing model group. All surgeries were performed by the same lead surgeon and employed a posterior approach to the hip joint.

### Nursing procedure

The nurse in charge of the project served as the research navigation nurse for this study, leading a workshop training session. Specific differences in perioperative nursing measures between the two groups are detailed in Table 1, with all other nursing measures adhering to the standard orthopedic perioperative care practices.

**Table 1:** Comparison of nursing measures between Traditional Care Group and ERAS Nursing Model Group

Nursing Measures	Traditional Care Group (n = 73)	ERAS Nursing Model Group (n = 87)
Education/Psychological Care	Notification of surgery and distribution of educational materials	Distribution of preoperative educational materials, guidance on use of commode and lung and postoperative exercises; dedicated care, with a focus on psychological support
Fasting	12 h for epidural, 8 h for water	6 h for epidural, 2 h for water, clear fluid 250 mL given 2 h before surgery
Pain Management	Analgesics as prescribed by the physician on a case-by-case basis	Celecoxib 0.2 g orally twice daily, PCA pump when necessary
Oxygen Therapy	None	Oxygen administered 3 times daily for 30 min
Warming Care	Single-layer cotton blanket transportation	Double-layer cotton blanket transportation, intraoperative warming blanket
Antithrombotic Care	Injection of low molecular weight heparin calcium	Oral rivaroxaban
Dietary Care	Fluid diet resumed 6 h postoperatively	Clear fluid 250 mL given 2 h postoperatively, fluid diet resumed based on patient’s response, dietitian-guided progression to normal diet
Functional Exercise	Based on patient’s subjective willingness, nurse-guided activities	Guided gradual exercises according to standardized electronic exercise prescription mode, emphasis on humanistic care, family accompaniment, joint guidance by family
Sleep Care	Mainly psychological care	Emphasis on sleep quality and duration, daily psychological care, administration of sleeping aids as prescribed when necessary

***Preoperative Routine Care***:

1. Vital signs were monitored;
2. Nurses assisted doctors in ensuring appropriate traction care;
3. Nurses helped patients attain a suitable position;
4. Patients were bathed one day prior to the surgery and skin was prepared as needed;
5. On the morning of surgery, patients were assisted in changing into surgical attire;
6. The anesthesia bed and all postoperative necessities were prepared.

***Postoperative Routine Care***:

1. Anesthesia care was selected based on the type of anesthesia used;
2. The patient was handed over from the anesthesiologist with a thorough understanding of intraoperative conditions and points of attention;
3. Changes in vital signs were observed;
4. Patients were helped to an appropriate position;
5. Care was provided for wounds and drainage tubes;
6. For patients with urinary catheters, proper catheter care was ensured;
7. Patients were educated on functional exercises and the procedure for first time out of bed.

### Observation indicators

The incidence of delirium at different time periods, sleep quality, time to first ambulation, length of hospital stays, and quality of life at different follow-up times were recorded and compared between the two groups. Delirium was diagnosed using the Confusion Assessment Method (CAM) at postoperative time points of 24 h, 48 h, and 72 h. CAM is a widely used instrument with high sensitivity and specificity [10, 11]. Sleep quality was assessed using the Pittsburgh Sleep Quality Index (PSQI) at postoperative intervals of 24 h, 48 h, and 72 h, with a higher score indicating poorer sleep quality [12]. Quality of life was evaluated at various postoperative time points (1, 3, 6, and 12 months) using the Harris score and SF-36 scale. Specifically, the Harris score and SF-36 were assessed at postoperative intervals of 1, 3, 6, and 12 months. The Harris Hip Score is a clinician-reported outcome measure specifically designed to evaluate various hip disabilities and methods of treatment in an adult population, with a possible score ranging from 0 (worst outcome) to 100 (best outcome). A higher Harris score and SF-36 composite score indicate better quality of life [13, 14].

### Statistical analysis

Statistical analysis was performed using SPSS 17.0 (IBM, USA). Before performing the t-test, the normality distribution of continuous data was evaluated using the Shapiro-Wilk test. Categorical data were presented as numbers and percentages, and Chi-square test was used for comparisons. Continuous data were presented as means ± standard deviations and compared using independent samples t-test. *P* < 0.05 was considered statistically significant.

## Results

Before delving into the demographic and clinical characteristics of the patients, it is essential to highlight the data’s normality. In our evaluation of the normality distribution of our continuous data using the Shapiro-Wilk test, the data was found to be normally distributed. This finding justified our use of the independent samples t-test for our subsequent analysis.

### Demographic and clinical characteristics

The demographics and clinical characteristics of the patients in the traditional care group and the ERAS nursing model group were compared. These included gender, age, level of education, fracture type, and surgical procedure. In both the traditional care group and the ERAS nursing model group, there were no significant differences in the gender distribution. The average age of patients was similar between groups. In terms of educational level, the groups were comparable, with no significant difference. Regarding fracture type, both groups had similar distribution of patients with Garden III and IV fractures. Finally, the type of surgery performed (total hip replacement vs. hemiarthroplasty) did not significantly differ between groups. These results suggest that the traditional care group and the ERAS nursing model group were well matched in terms of demographic and clinical characteristics, allowing for a fair comparison of the impact of the different postoperative care strategies. The distribution of these characteristics is shown in Table 2.

**Table 2:** Comparison of preoperative general data between Traditional Care Group and ERAS Nursing Model Group

Characteristics	Traditional Care Group (n = 73)	ERAS Nursing Model Group (n = 87)	t/χ2 value	*P*-value
Gender			0.366	0.546
- Male	36	42		
- Female	37	45		
Age (x ± s, years)	72.4 ± 3.3	73.2 ± 3.6	-1.520	0.134
Level of Education			1.520	0.680
- Illiterate	27	38		
- Primary School	42	38		
- Junior High School	9	10		
- High School	0	1		
Fracture Type			0.030	0.868
- Garden III	30	38		
- Garden IV	43	49		
Type of Surgery			0.995	0.322
- Total Hip Replacement	50	57		
- Hemiarthroplasty	23	30		

### Incidence of postoperative delirium

The comparison of the incidence of delirium between the two groups of patients always showed a statistically significant difference postoperatively, except for the first 24 h (*P* < 0.05), as detailed in Table 3.

**Table 3:** Comparison of postoperative delirium incidence between Traditional Care Group and ERAS Nursing Model Group

	Traditional Care Group (n = 73)	ERAS Nursing Model Group (n = 87)	χ² value	*P*-value
Postoperative 24 h	4	2	1.674	0.196
Postoperative 48 h	7	3	3.991	0.046
Postoperative 72 h	5	2	7.078	0.008
Total cases	17	7	8.052	0.005

### Duration of Hospital Stay and Time to First Ambulation

Significant differences were also observed between the two groups in terms of the duration of hospital stay and the time to first ambulation postoperatively (*P* < 0.05), as shown in Table 4.

**Table 4:** Comparison of hospital stay duration and first postoperative ambulation time between Traditional Care Group and ERAS Nursing Model Group

	Traditional Care Group (n = 73)	ERAS Nursing Model Group (n = 87)	t-value	*P*-value
Length of Hospital Stay (days, mean ± SD)	8.5 ± 2.5	7.3 ± 3.4	2.86	0.005
Time to First Ambulation Postoperatively (days, mean ± SD)	5.7 ± 1.3	4.0 ± 2.9	4.96	< 0.001

### Sleep quality postoperatively

Significant differences were observed in sleep quality between the two groups at 24, 48, and 72 h postoperatively (*P* < 0.05), as shown in Table 5.

**Table 5:** Comparison of postoperative sleep quality between Traditional Care Group and ERAS Nursing Model Group

	Traditional Care Group (n = 73)	ERAS Nursing Model Group (n = 87)	t-value	*P*-value
Postoperative 24 h	15.5 ± 4.0	13.8 ± 2.7	3.3934	< 0.001
Postoperative 48 h	13.5 ± 5.3	10.8 ± 4.3	3.9231	< 0.001
Postoperative 72 h	11.9 ± 2.3	8.2 ± 2.1	12.1346	< 0.001

### Quality of life postoperatively

In terms of postoperative quality of life, the ERAS nursing model group demonstrated higher scores on the Harris Hip Score and the SF-36 scale during follow-up visits than the traditional care group, indicating a statistically significant difference (*P* < 0.05), as illustrated in Table 6.

**Table 6:** Comparison of postoperative quality of life between Traditional Care Group and ERAS Nursing Model Group

	Postoperative 1 month	Postoperative 3 months	Postoperative 6 months	Postoperative 12 months
Harris Score				
Traditional Care Group (n = 73)	66.4 ± 9.7	70.1 ± 6.1	72.9 ± 2.1	76.9 ± 2.5
ERAS Nursing Model Group (n = 87)	68.2 ± 5.6	72.9 ± 4.4	77.6 ± 4.3	82.6 ± 7.7
t value	-1.189	-2.618	-6.917	-5.562
*P* value	< 0.001	< 0.001	< 0.001	< 0.001
SF-36 Score				
Traditional Care Group (n = 73)	67.6 ± 8.2	71.0 ± 8.7	75.1 ± 5.1	79.5 ± 2.8
ERAS Nursing Model Group (n = 87)	72.1 ± 5.6	75.0 ± 6.5	79.1 ± 9.7	85.4 ± 11.7
t value	-2.145	-1.567	-8.467	-6.967
*P* value	< 0.001	< 0.001	< 0.001	< 0.001

## Discussion

Our study delineated several pivotal findings regarding the effectiveness of the ERAS nursing model compared to the traditional care group in the context of postoperative care. To begin, both the traditional care group and the ERAS nursing model group were found to be well-matched in terms of demographic and clinical characteristics. This ensures a balanced foundation for comparing the impact of the two distinct postoperative care strategies. A crucial finding of our research was the marked difference in the incidence of postoperative delirium between the groups, excluding the initial 24 h post-operation. Delirium, particularly postoperative, has been identified as a significant concern, primarily due to its association with prolonged hospitalization, increased morbidity, and augmented healthcare costs. The lesser incidence in the ERAS nursing model group underscores the potential benefits of this approach in mitigating postoperative complications. Further, our study shed light on the expedited timeline to first ambulation and shorter hospital stays in the ERAS group. Prolonged bed rest post-surgery can predispose patients to complications such as venous thromboembolism and pressure ulcers. By contrast, early ambulation can enhance functional recovery, reduce muscle atrophy, and minimize the risk of postoperative complications.

Furthermore, shorter hospital stays not only reduce healthcare costs but also minimize the risk of hospital-acquired infections and contribute to patient satisfaction. Sleep quality, an often overlooked but crucial determinant of postoperative recovery, also exhibited significant variations between groups. Optimal sleep is linked with better pain control, enhanced mood regulation, and improved cognitive and physical functions. The superior sleep quality in the ERAS group could potentially influence these domains, leading to improved overall outcomes. Lastly, the discernible difference in postoperative quality of life, as measured by the Harris Hip Score and the SF-36 scale, speaks volumes about the advantages of the ERAS nursing model. Quality of life is an all-encompassing measure, reflecting not only physical but also psychological and social facets of well-being. Our findings suggest that the ERAS model might hold promise in bolstering postoperative quality of life.

In conclusion, our results underline the potential merits of the ERAS nursing model in optimizing postoperative outcomes. As healthcare evolves, such models that amalgamate efficiency with enhanced patient care could represent the future of postoperative management.

Building on these broad observations, a deeper dive into specific statistical findings further elucidates the differential impact of the ERAS model compared to traditional care. Our research findings revealed statistically significant differences in the number of incidences 48 and 72 h post-surgery between the ERAS group and the traditional care group (*P* < 0.05). However, no statistical significance was observed in the number of incidences 24 h post-surgery between the two groups (*P* > 0.05). This suggests that the benefits of ERAS become more noticeable as the recovery progresses but may not be immediately evident within the first 24 h post-surgery. A possible explanation for this may be the sustained analgesic effect of the anesthetic drugs administered during surgery, which might be a confounding factor in the early postoperative period [15, 16]. Significant differences were also observed in the quality of sleep between the two groups (*P* < 0.05). As discussed earlier, this improvement in sleep quality is critical in reducing delirium incidence, improving overall rehabilitation, and promoting early return to normal activities. Additionally, the ERAS group showed significantly earlier first out-of-bed times and shorter hospital stays compared to the traditional group (*P* < 0.05). These findings indicate that the ERAS model effectively accelerates postoperative recovery, minimizing the adverse effects associated with prolonged bed rest and hospitalization. Furthermore, higher scores on the Harris Hip Score and SF-36 scale were noted in the ERAS group during follow-up, implying better functional outcome and overall quality of life compared to those receiving traditional care (*P* < 0.05). These measures, coupled with the noticeable reduction in delirium incidence, attest to the potential of the ERAS model to significantly improve postoperative recovery and patient quality of life.

### Optimization of preoperative preparation

Change in Preoperative Patient Education Approach. In the ERAS group, bedside nurses provide comprehensive preoperative education about the disease course to the patient, demonstrating patient-centered care and cooperative treatment concepts. This approach improves the nurse’s attention to the patient’s psychological health, fosters a closer understanding between the nurse and the patient, and reduces the trust distance [17, 18].

Changes in Preoperative Fasting Guidelines. Following the guidelines and research by FRASSANITO et al. [19], the ERAS group adopted a 6-hour fasting and 2-hour fluid restriction regimen. This reduced fasting period helps alleviate insulin resistance and increased catabolism caused by hunger, which alleviates postoperative intestinal edema and facilitates early resumption of food intake. In comparison, traditional practices involve longer periods of fasting, increasing discomfort and anxiety, inducing endocrine stress response, maintaining high blood sugar levels, and causing a state of irritability, which are unfavorable for postoperative recovery [20].

Change in Preoperative Intervention Approach. Brain tissues are sensitive to hypoxia, and a decrease in acetylcholine synthesis and release, along with an imbalance between oxygen supply and consumption, can induce brain damage and delirium [21]. Elderly patients can often tolerate long-term hypoxic conditions without apparent symptoms, so conventional treatments providing oxygen therapy intervention when patients feel discomfort are often delayed. The ERAS group provides oxygen therapy upon admission, which could potentially reduce the occurrence of delirium and improve the patient’s quality of life.

### Optimization of intraoperative care

Intraoperatively, hypothermia can increase sympathetic nerve tension, causing peripheral vasoconstriction, increased blood viscosity, increased cardiovascular and cerebral load, and increased incidences of shivering and restlessness, potentially leading to arrhythmia and coagulopathy. Using a warming blanket during surgery to reduce the loss of body temperature helps protect basic organ functions, decrease the occurrence of delirium, and assist in recovery.

### Optimization of postoperative care

Changes in Pain Management Philosophy. The traditional group mainly uses self-controlled analgesic pumps and intermittent opioid analgesics postoperatively, which are often passive measures, yielding modest results and potent side effects [22, 23]. In contrast, the ERAS group incorporates the concept of preventive analgesia, providing routine use of celecoxib until the third postoperative day [24, 25]. Starting from the preoperative period, the cumulative analgesic effect offers preemptive analgesia, attains steady-state blood drug concentrations early postoperatively, and alleviates pain while improving body function. Combining correct assessment and low-dose opioids can achieve equivalent analgesic effects.

Changes in Exercise Philosophy. The ERAS group advances patient functional exercise to the preoperative period and devises individualized exercise prescriptions based on the patient’s condition. Early ambulation, coupled with the use of rivaroxaban, encourages muscle exercise, and accelerates blood circulation, thereby preventing thrombosis [26, 27]. As evident in Tables 4, 5 and 6, this approach has yielded positive clinical results, significantly reducing bed rest duration, decreasing complications, indirectly shortening the patient’s hospital stay, improving patient mobility, and enhancing the quality of life, bringing tangible benefits to both patients and their families.

Changes in Sleep Attention. The ERAS group places significant emphasis on understanding the patient’s sleep conditions. As shown in Table 5, there was a marked improvement in sleep quality. Improvements in insomnia can aid in alleviating postoperative pain, promote early ambulation and functional exercise, and increase patient satisfaction.

ERAS has been widely adopted across various medical fields due to its promising impacts on postoperative outcomes. Jiang et al [7]. emphasized precision nursing under ERAS, underscoring areas like assessment and information technology. Notably, different studies have approached ERAS from unique angles, tailoring their research based on specific outcomes or patient demographics. Our research, converging on ERAS’s benefits, delves deeper into outcomes like delirium and rehabilitation in elderly femoral neck fracture patients. Kim et al. [8] highlighted ERAS’s efficacy in shortening hospital stay post-thoracolumbar surgery. While both studies endorse ERAS, our focus on delirium and sleep quality offers nuanced insights into fracture care. Pang et al. [28] combined WeChat preaching with ERAS, assessing limb motor function and complications. Their innovative approach demonstrates the adaptability of ERAS principles in diverse contexts. While their integration of WeChat is groundbreaking, our study’s specificity addresses the needs of an elderly fracture demographic. Furthermore, Li et al. [29] used model-based analysis to evaluate ERAS’s impact in surgical settings, accentuating team awareness. Aligning with their broader ERAS support, our study narrows its lens on outcomes such as sleep and postoperative life quality. Building on this extensive groundwork, our research aims to further enrich and expand the understanding of ERAS’s potential, particularly in the realm of fracture care.

While our findings offer valuable insights, our study has limitations. The sample size was relatively small, and potential biases could arise from varying nursing capabilities. We did not calculate the Cronbach’s alpha for our scales, focusing instead on clinical outcomes. Though these scales have established reliability, not assessing their internal consistency in our sample is a limitation. Additionally, potential links between perioperative hypoalbuminemia, occult postoperative bleeding, and delirium warrant further investigation.

## Conclusions

Our findings affirm that the ERAS nursing model can potentially improve postoperative sleep quality, reduced delirium occurrence, shortened average hospitalization duration, and enhanced patients’ quality of life in elderly patients with femoral neck fractures. However, more extensive, and rigorous studies are required to further validate these findings and explore other associated factors.

## Data Availability

The datasets used and/or analyzed during the present study are available from the corresponding author on reasonable request.
